# Neuroinflammatory Mechanisms of Mitochondrial Dysfunction and Neurodegeneration in Glaucoma

**DOI:** 10.1155/2021/4581909

**Published:** 2021-04-15

**Authors:** Joao N. Duarte

**Affiliations:** ^1^Neuroinflammation Unit, Biotech Research & Innovation Center, University of Copenhagen, Copenhagen, Denmark; ^2^Department of Ophthalmology, Rigshospitalet, Copenhagen, Denmark; ^3^Department of Clinical Immunology, Section 7631, Rigshospitalet, Copenhagen, Denmark; ^4^Department of Drug Design and Pharmacology, University of Copenhagen, Copenhagen, Denmark

## Abstract

The exact mechanism of retinal ganglion cell loss in the pathogenesis of glaucoma is yet to be understood. Mitochondrial damage-associated molecular patterns (DAMPs) resulting from mitochondrial dysfunction have been linked to Leber's hereditary optic neuropathy and autosomal dominant optic atrophy, as well as to brain neurodegenerative diseases. Recent evidence shows that, in conditions where mitochondria are damaged, a sustained inflammatory response and downstream pathological inflammation may ensue. Mitochondrial damage has been linked to the accumulation of age-related mitochondrial DNA mutations and mitochondrial dysfunction, possibly through aberrant reactive oxygen species production and defective mitophagy. The present review focuses on how mitochondrial dysfunction may overwhelm the ability of neurons and glial cells to adequately maintain homeostasis and how mitochondria-derived DAMPs trigger the immune system and induce neurodegeneration.

## 1. Introduction

Glaucoma is a complex and multifactorial neurodegenerative disease characterized by the irreversible loss of retinal ganglion cells' (RGCs) soma and degeneration of the optic nerve axons [1]. Glaucoma is the most common optic neuropathy and the leading cause of irreversible blindness worldwide [2, 3]. It is generally accepted that elevated intraocular pressure (IOP) is a major risk factor [2].

The exact mechanisms by which elevated IOP triggers axonal degeneration and RGC death are yet to be known. IOP-induced mechanical compression to the optic nerve head (ONH), at the level of the lamina cribrosa, might directly lead to ischemia-hypoxia damage, blockage of axonal transport, and deprivation of growth factors [4–7]. However, indirectly, damage to RGCs may also result from the action of factors released by activated glial cells located in the lamina cribrosa [8–11]. Regardless of the mechanisms that initiate damage, there is evidence that, in glaucoma, these events converge into axonal degeneration, RGC death, and clinical overlap between the different glaucoma subphenotypes.

Notwithstanding the major role of IOP in the progression of glaucoma, in patients with normal-tension glaucoma (NTG), the elevation of IOP is not necessary for the development of glaucomatous damage [12]. Moreover, the reduction of IOP does not always prevent neurodegeneration, and many patients progress with the disease despite having IOP within normal range [13, 14]. This suggests that mechanisms other than IOP biomechanical and/or ischemic injury may be responsible for the neurodegenerative process. Although the precise mechanisms that lead to RGC insult and loss have not been identified, accumulating evidence supports a primary role of inflammation and the immune system [15–17]. Despite these indications, ocular hypertension remains the only target for current glaucoma therapies.

The central nervous system (CNS), which also includes the retina and optic nerve, is an immune-privileged site where immune functions are tightly regulated and are mediated by a limited number of cell types [18]. The onset of inflammation in glaucoma is hypothesized to be triggered by an altered crosstalk between RGCs and glial cells that involves the release of proinflammatory mediators, such as reactive oxygen species (ROS), nitric oxide (NO), tumor necrosis factor-alpha (TNF-*α*), and interleukin-1*β* (IL-1*β*) [9, 19–23]. Despite the recognized role of inflammation in glaucoma, to date, the key inflammatory signals and events that lead to polarization of microglia and astrocytes in the disease progression are not known. Transcriptomic studies showed that many inflammatory genes are upregulated in the retina and ONH in the early stage of the disease [17, 24, 25]. Some of the pathogenic events in glaucoma have been attributed to modifications of neurotrophin and glutamate signaling, excitotoxicity, oxidative stress, mitochondrial dysfunction, protein misfolding, hypoxia, ischemia, autoimmunity, and autophagy dysfunction [26–28].

The present review focuses on the role of the immune system in glaucomatous neurodegeneration and its contribution to disease progression. In particular, it discusses how mitochondrial dysfunction and concomitant neuronal and glial pathological processes can induce and perpetuate sterile inflammation in glaucoma, and which crossroads may be implicated in that. In the first part of the review, potential causes of neuronal mitochondrial dysfunction are addressed. In the second and third parts, the discussion will focus on currently known mechanisms of neuroglial dysfunction and the immune response secondary to mitochondrial damage.

To obtain a comprehensive collection of publications dealing with the neurodegenerative inflammatory response in glaucoma, multiple unrestricted PubMed searches specifying the occurrence of the term “glaucoma” in combination with “inflammation,” “neurodegeneration,” “immune response,” “mitochondrial dysfunction,” “oxidative stress,” “mitophagy,” and “mutation” were performed. The search was limited to primary open-angle glaucoma (POAG). Human studies that dealt with primary closed-angle glaucoma or secondary glaucoma were not considered for this review.

## 2. Mitochondrial Dysfunction in Retinal Ganglion Cells

The retina is one of the most metabolically active tissues in the body and requires a precise regulation of energy production to meet its consumption needs [29, 30]. Energy in the form of adenosine triphosphate (ATP) is required to synthesize neurotransmitters, organize synaptic vesicles, restore ion gradients, buffer calcium, and transport cargo bidirectionally along axons [30, 31]. Due to the absence of saltatory conduction, the unmyelinated portion of RGC axons within the retina requires more energy for the generation of action potentials [32]. In response to this high metabolic demand, a large proportion of mitochondria populate the unmyelinated portion of RGC axons [32–35].

Strict coordination between mitochondrial biogenesis, dynamics, transport, and degradation is essential to preserve the integrity of mitochondria within RGCs [30, 35]. These events are tightly regulated to ensure that mitochondria can adapt to fluctuations in energy requirements [36, 37]. Even though these metabolic processes are not unique to RGCs, these cells have lower tolerance for mitochondrial damage, and an inadequate supply of healthy mitochondria or the accumulation of defective mitochondria may be the origin of an energy crisis in RGCs [28, 30]. Among other potential causes, mitochondrial dysfunction has been linked to oxidative stress, mutations in mitochondrial DNA (mtDNA), and deficient mitophagy [38, 39].

### 2.1. Oxidative Stress

Oxidative stress is broadly defined as an imbalance that favors the production of ROS over antioxidant defenses. A consequence of electron transport through mitochondrial oxidative phosphorylation (OXPHOS) complexes is the generation of ROS, such as superoxide anions (O_2_^−^) and hydrogen peroxide (H_2_O_2_). Although ROS are key second messengers in various redox-sensitive signaling pathways, they can damage cellular proteins, lipids, and nucleic acids via oxidation [40]. As mitochondria are a significant source of ROS in many eukaryotic cells, especially in the context of age-related deterioration of mitochondrial electron chain transfer, mitochondrial ROS have been suggested to be an important immunostimulatory stimulus in glaucoma [40, 41].

Oxidative stress occurs as a result of malfunctions in one or more of the mitochondria's four main functions: generation of energy in the form of ATP; regulation of ROS production; regulation of cytosolic calcium levels; and modulation of apoptosis via mitochondrial permeability [42, 43]. In normal conditions, the generation of ROS in low levels is blocked by antioxidants, such as glutathione peroxidase, superoxide dismutase, and catalase. The most active sites of mitochondrial ROS production (i.e., sites of electron leakage) are complex I and complex III of the OXPHOS chain [44]. Although these sites do not constitute a major source of ROS within the cell, the damage inflicted by mitochondrial ROS can be very detrimental due to a particular vulnerability of the mitochondria to oxidative stress, notably to mtDNA. ROS, when in excess, can also induce lipid peroxidation and apoptosis by increasing mitochondrial membrane permeability and by inhibiting the mitochondrial respiratory chain [45–48].

The accumulation of neurotoxic levels of glutamate is another effect of oxidative stress. Glutamine synthase, which converts retinal glutamate into a nontoxic form, and glutamate transporter proteins were modified by ROS in experimental models of ocular hypertension [49–51]. Many of these cellular pathways and components that are damaged by oxidative stress become themselves the cause of further ROS production, perpetuating a cycle of cellular injury.

The cellular accumulation of ROS, regardless of the triggering stress condition, can mediate the induction of autophagy [52]. Autophagy is a nonselective degradation pathway that primarily promotes cellular protection through clearance of damaged organelles and protein aggregates. The autophagic process is fundamental to neuronal homeostasis and can be distinguished into more organelle-specific pathways, such as mitophagy for selective removal of mitochondria (see Section 2.3). In RGCs, ROS serve as signaling molecules in the induction of autophagosomes by regulating the activity of the cysteine protease ATG4, which seems to be a key element in the activation of autophagy [53]. Yoshimura et al. found that mRNA levels of ATG4B are high in brain tissues and retina, among other sites [54]. Dysfunction of autophagy has also been linked to brain chronic neurodegenerative diseases, suggesting common mechanisms of autophagy-related neuronal loss [52].

**Figure 1:**
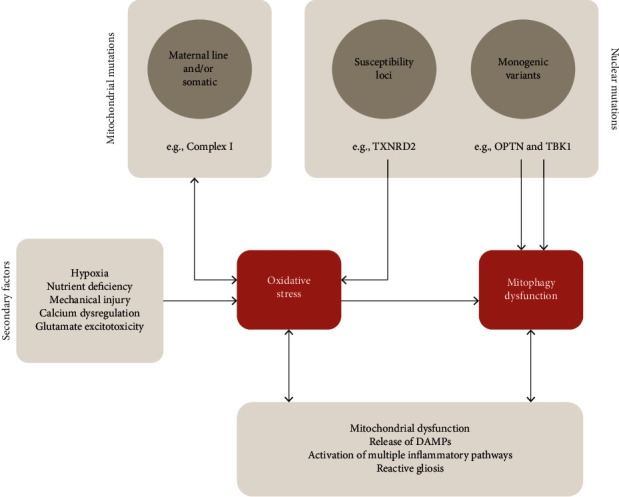
Mechanisms of mitochondrial dysfunction in retinal ganglion cells (RGCs). Mutations in mitochondrial and nuclear genomes can cause mitochondrial dysfunction via oxidative stress or defective mitophagy. Oxidative stress can also be generated through secondary causes external to the mitochondria, such as hypoxia. Mitochondrial-derived components can act as damage-associated molecular patterns (DAMPs) and trigger multiple inflammatory pathways that cause reactive gliosis and RGC loss.

A long list of factors has been linked to ROS formation in glaucoma. To some extent, these factors seem to be related to intrinsic abnormal function of mitochondria (primary) or to external events that expose the RGCs and neuroglial mitochondria to stress (secondary), such as compromised blood flow, hypoxia, nutrient deficiency, calcium dysregulation, or mechanical injury (Figure 1) [49, 55–59].

Oxidative stress in POAG has been linked to specific OXPHOS defects, in particular to defects in complex I. Using a lymphoblast-based model to measure systemic mitochondrial function, Lee et al. and Van Bergen et al. showed, in two different glaucoma patient cohorts, decrease rates of ATP synthesis related to complex I defects [60, 61]. Although those defects had a low impact on the in vitro growth of lymphoblasts, they possibly rendered RGCs more susceptible to oxidative stress and metabolic crisis due to the high energetic requirements of RGCs and the presence of multiple cellular stressors in glaucoma. These results are in line with similar findings for complex I defects in hereditary optic neuropathies and brain neurodegenerative diseases [62, 63].

Oxygen and nutrient deficits not only cause RGCs to become bioenergetically compromised but also dramatically increase ROS production. This increase is due to slower electron transportation in the OXPHOS, which augments the reduction state of electron carriers and favors superoxide production at low oxygen concentrations [64]. Studies have also shown the upregulation of hypoxia-inducible factor 1 (HIF1) in glaucoma as a cellular response to reduced oxygen levels in the retina and ONH [65]. HIF1-alpha is a transcriptional activator that induces the expression of several proteins whose main function is to increase oxygen availability in hypoxic tissues [43]. These findings suggest the importance of hypoxia signaling mechanisms in the pathogenesis of glaucoma.

Oxidative stress has also been linked to abnormal cellular calcium influx [66]. Even though calcium serves a major role in normal intracellular metabolism and signaling (including the generation of ROS), an uncontrolled influx may occur through a variety of insults, such as loss of cell membrane integrity and inhibition of calcium ATPase activity [67]. Once the cell has been exposed to an excess of calcium, a synergic effect between calcium and ROS may occur, as ROS can also inhibit calcium ATPase activity in neurons [68]. An excessive influx of calcium and neuronal damage has also been linked to homocysteine accumulation. The mechanism of this homocysteine-induced RGC toxicity seems to take place through direct overstimulation of N-methyl-D-aspartate (NMDA) receptors and a subsequent increase in intracellular calcium and formation of ROS [69].

In addition to retinal and optic nerve damage caused by oxidative stress, the trabecular meshwork in the anterior segment of the eye is also vulnerable to ROS [70, 71]. This event, which has been connected in some cases to genetic mutations in myocilin, may lead to increased resistance of the aqueous humor outflow and cause further retinal exposure to high IOP [71–73]. Mechanical injury to the ONH caused by increased IOP induces neuronal oxidative stress and lipid peroxidation, which increments the risk of neurodegeneration [49, 74, 75]. In a study conducted in aged mice by Kong et al., even short-term elevations of IOP were shown to significantly increase oxidative stress [76].

Increasing evidence also points out the impact of oxidative stress and mitochondrial dysfunction in Müller cells, which may contribute to the pathogenesis neurodegenerative conditions in the retina. As Müller cells have numerous supporting functions to maintain RGC homeostasis, with many of them being metabolic-related, the impact of restricted energy availability in Müller cells has been implicated in glaucomatous RGC loss [77, 78]. A published review by Toft-Kehler et al. on mitochondrial function in Müller cells can provide an in-depth discussion on the topic [28].

As oxidative stress also represents a failure of endogenous antioxidant defenses to meet an increase of ROS, many studies have been demonstrating an accumulation oxidative stress markers in the serum, aqueous humor, and retina of glaucoma patients, as well as suggesting a general compromise of antioxidant defense in these patients [41, 48, 49, 75, 79, 80]. In a case-controlled study conducted by Yuki et al., the incidence of NTG was significantly correlated with high serum total antioxidant levels and low urinary 8-hydroxy-2′-deoxyguanosine/creatinine (a marker of DNA damage from oxidative stress), suggesting systemic oxidative stress [81]. Similarly, a long prospective study in a cohort of 3500 patients with glaucoma showed an association between low intake of antioxidant nutrients and a higher risk of POAG [82]. Identical results have been also documented in experimental models of glaucoma [83].

Several works have assessed the impact of antioxidant treatment in the reduction of oxidative stress in RGCs. Brimonidine, an alpha-adrenergic receptor agonist currently used to lower IOP, has been found to have a neuroprotective effect beyond IOP lowering [84–86]. Brimonidine seems to prevent abnormal elevations of cytosolic calcium and RGC apoptosis through mechanisms not yet fully elucidated [85]. Tempol, a nitroxyl antioxidant, resulted in increased survival of RGCs exposed to TNF-*α* and hypoxia in the presence of a caspase inhibitor. Crocin, a carotenoid, has antioxidant properties that are capable of suppressing ROS production, increasing mitochondrial membrane potential, and enhancing RGC viability upon H_2_O_2_ treatment [45, 87]. Coenzyme Q10 and N-acetyl cysteine are antioxidant compounds that directly target mitochondria and have shown beneficial effects in experimental models of glaucoma. Coenzyme Q10, as an essential cofactor in mitochondrial OXPHOS, afforded retinal protection in an ischemia-reperfusion rat model [88]. Similarly, the administration of N-acetyl cysteine, which enhances mitochondrial OXPHOS, decreased oxidative stress in a rat model of ocular hypertension [84].

The incorporation of antioxidants into mitochondrial membranes has also been shown to prevent lipid peroxidation, a process known to be initiated by ROS that can lead to cell membrane damage and RGC loss. In a rabbit model of glaucoma, 10-(6′-plastoquinonyl) decyltriphenylphosphonium (SkQ1), a mitochondrial-targeted plastoquinone-containing antioxidant, was able to reverse signs of glaucomatous injury [47]. Likewise, in a mouse model of glaucoma, choric acid and 3,5-dicaffeoylquinic acid of ethanol extract of *Crepidiastrum denticulatum* seemed to reduce lipid peroxidation and protect against RGC death [89]. Yokota et al. also showed that molecular hydrogen, through peroxynitrite scavenging capacity, protected lipid peroxidation and prevented retinal cell apoptosis [69].

### 2.2. Mitochondrial DNA Mutations

Glaucoma has not been clearly linked to nuclear genome mutations that result in mitochondrial dysfunction [90]. Large genome-wide association studies (GWAS) identified, with limited success, susceptibility loci associated with mitochondrial dysfunction [60, 61, 91]. A large meta-analyzed GWAS conducted by Bailey et al. identified, for example, a single nucleotide polymorphism in thioredoxin reductase 2 (TXNRD2), a mitochondrial protein required for redox homeostasis [92]. The effects of TXNRD2 in vivo were reported by Caprioli et al. who observed reduced RGC death after optic nerve axotomy in experimental models of glaucoma overexpressing TXNRD2 when treated with pharmacologically induced oxidative stress [93].

Mutations with Mendelian inheritance have only been associated with rare cases of POAG with early disease onset. These monogenic variants have been identified in myocilin (MYOC), optineurin (OPTN), and TANK binding kinase 1 (TBK1) [94–99]. Mutations in MYOC, as mentioned above, primarily cause oxidative stress abnormalities in the trabecular meshwork leading to increased IOP. Mutations in OPTN and TBK1 have been linked to dysregulation of mitophagy and apoptosis, and activation of inflammation with RGC loss (Figure 1). A more detailed discussion about the role of OPTN and TBK1 mutations in POAG is provided in the next section.

Several attempts to sequence and identify mutations in mitochondrial DNA have been hindered by mtDNA heteroplasmy, i.e., the multiple variants of mtDNA that can be present within a cell or a tissue. However, massive parallel sequencing tools have allowed better recognition of large deletions in the mitochondrial genome in a few studies using small cohorts of patients with glaucoma [100–102]. Even though these studies extrapolated mitochondrial variants from peripheral blood leukocytes, they provide evidence of an association between systemic mtDNA defects and glaucoma. Moreover, from peripheral blood leukocytes, Sundaresan et al. identified mtDNA mutations in complex I in approximately one-third of POAG patients [102]. These results are in line with previous studies that addressed the possibility of defects in complex I resulting in impaired respiration rates and ATP production in lymphocytes of patients with POAG [60, 61]. To what proportion mtDNA variants are inherited through maternal line or acquired throughout life as somatic mutations is currently not known.

Somatic mutations are prone to accumulate more frequently in the mitochondrial genome due to the lack of protective histones and an efficient DNA repair system associated with the nuclear genome [103]. Mitochondrial abnormalities are associated with a number of optic neuropathies, and accumulating evidence indicates that age-related mitochondrial defects play a central role in the pathogenesis of glaucoma [44, 104, 105]. Mitochondrial genetic variants have also been linked to other neurodegenerative diseases, including Parkinson's disease and Alzheimer's disease, which are thought to have some overlapping pathologic features with glaucomatous neurodegeneration [106–109].

Exposure of mtDNA to ROS can induce its degradation, and mtDNA degradation products are found in human cerebrospinal fluid and plasma [110]. Transfection of mouse primary astrocytes with degraded mitochondrial polynucleotides was shown to cause a proinflammatory response, which was characterized by the upregulation of TNF-*α*, IL-1*β*, IL-6, and monocyte chemotactic protein 1 [110]. This observation suggests a mechanism for mtDNA degradation and downstream activation of proinflammatory phenotypes in glial cells (Figure 1). It was also previously reported that mtDNA deteriorates in response to hydrogen peroxide in HA-1 hamster ovarian cells, an effect that was not observed with nuclear DNA or cytoplasmic RNA [111]. Degradation of the mitochondrial genome was apparent in both mtDNA and mitochondrial RNA species.

Even though an immunostimulatory release of mtDNA may occur through the formation of mitochondrial-derived vesicles or necrotic cell death, a more controlled and less immunogenic mechanism of mtDNA processing has also been proposed [40, 112]. PTEN-induced kinase 1 (PINK1) and Parkin, two mitochondrial proteins linked to mitophagy and Parkinson's disease, have been shown to actively inhibit mitochondrial-derived vesicles and mitochondrial antigen presentation in favor of mitophagy [112]. This suggests that impairments in PINK1-Parkin signaling may contribute to inflammation in neurodegenerative conditions.

### 2.3. Deficient Mitophagy

Mitophagy is the process in which damaged mitochondria are degraded by the autophagy system. This process is vital for removing debris and aggregating proteins from the cells, thereby protecting cells of potential cell-damaging proteins. For postmitotic cells like neurons, mitophagy is an essential survival mechanism for neuroprotection and elimination of toxic compounds. Damaged mitochondria are singled out and degraded, through the activities of PINK1 and Parkin [113].

In physiological conditions, PINK1, imported from the cytosol via mitochondrial translocases, is usually found within the inner mitochondrial membrane, where it is exported, proteolytically cleaved, and degraded. PINK1 has a mitochondrial targeting sequence that directs the protein into the correct mitochondrial subcompartment [114]. During mitochondrial damage, the mitochondrial membrane potential is altered and PINK1 is prevented from entering through the outer mitochondrial membrane (OMM) [114]. As PINK1 molecules accumulate on the OMM, they start a cascade of events to recruit and activate cytosolic Parkin. Parkin initiates the process of degradation of the mitochondrion, which involves ubiquitination of the mitochondria, autophagosome engulfment, and lysosomal fusion. Both Parkin and PINK1, therefore, contribute to the build-up of phosphorylated ubiquitin and abundant accumulation of PINK1 on the OMM, which acts as an indicator to the cell where the mitochondrion is damaged and will need to be removed [115].

Mitophagy can be activated by ROS in response to various conditions, such as oxidative stress, starvation, and mechanical injury [52, 116]. Wang et al. showed that ROS have a direct effect in PINK1-Parkin signaling and these effects can be reversed with antioxidant treatment using superoxide dismutase-2 [116]. Independently from the direct effects of ROS in mitophagy, autosomal recessive forms of glaucoma, familial Parkinson's disease, and amyotrophic lateral sclerosis (ALS) have been associated with dysfunctions of PINK1-Parkin pathway.

Specific mutations in OPTN and TBK1 have been implicated in the pathogenesis of a subgroup of NTG with early onset (Figure 1) [99]. OPTN is a ubiquitously expressed protein involved in neuroinflammation, Golgi maintenance, vesicular trafficking, and autophagy. In mitophagy, OPTN acts as a receptor protein, downstream of PINK1-Parkin, that is translocated to damaged mitochondria via binding to ubiquitinated mitochondrial proteins and through interaction with microtubule-associated protein 1 light chain 3 (LC3) to couple the mitochondria with an autophagosome for degradation [99, 117]. Several hypotheses about the role of OPTN in glaucoma have indicated a neuroprotective function and shown that mutations may lead to RGC loss through downregulation or dysfunction of this protein. Due to a relatively high expression of OPTN in the retina, mutations in this protein may increase RGC vulnerability. Chernyshova et al. showed that several glaucoma OPTN-mutant proteins (E50K, A377T, H486R, H26D, E103D, T202R, and A336G) seemed to restore mitophagy in HeLa cells where Parkin-dependent mitophagy was previously inhibited [117]. Such results were not seen in OPTN-mutant proteins associated with ALS, which suggests that glaucoma associated with OPTN mutations may occur through a mechanism independent from mitophagy dysfunction. A different study, conducted by Shim et al., found that acute overexpression of OPTN E50K mutant in rat RGCs caused increased mitophagy, ROS production, and activation of apoptosis via Bax pathway [118]. A possible explanation for an increased mitophagy was elucidated by reports that observed that the OPTN E50K and M98K mutants displayed striking affinity to TBK1 [119–121]. As TBK1 stimulates autophagy by phosphorylating and activating OPTN, abnormal TBK1 activation of OPTN may explain the increase in mitophagy [98]. Minegishi et al. also reported that the OPTN E50K mutant forms aggregates of insoluble protein in neuronal cells derived from induced pluripotent stem cells, which seem to lead to cell death [121].

Copy number variation mutations of the TKB1 gene have also been found in a subgroup of NTG [98]. A duplication of TBK1 has been found to increment TBK1 transcription, which is linked to a gain of function role of TBK1. Transgenic mice that had the TBK1 gene duplicated or triplicated showed progressive loss of RGCs, proportional to the number of gene copies, with no increase in IOP [122]. Apart from OPTN phosphorylation, TBK-1 is also able to activate mitophagy in an OPTN-independent manner through p62 phosphorylation, suggesting that OPTN and p62 regulate mitophagy by different mechanisms [123].

Apart from a direct role of TBK1 and OPTN in mitophagy, both proteins are also involved in innate immune inflammatory signaling, such as through the activation of nuclear factor-kappa B (NF-*κ*B) [124]. NF-*κ*B is a family of transcription factors that are key regulators of cytokine production and respond to a wide variety of inflammatory stimuli.

The role of OPTN in NF-*κ*B regulation has been somewhat controversial due to divergent results that came from different cell types and stimuli [125, 126]. OPTN was shown to interact with cylindromatosis (CYLD), an enzyme that deubiquitinates receptor-interacting protein (RIP) to inhibit TNF-*α*-induced NF-*κ*B activation [127]. Studies with the glaucoma-associated OPTN H486R mutant demonstrated loss of interaction of OPTN and CYLD, which resulted in increased NF-*κ*B signaling induced by inflammatory cytokines [128, 129]. Tanishima et al. also found another binding partner of OPTN, which is involved in NF-*κ*B signaling [128]. The interaction of OPTN with IL-1*β* receptor-associated kinase 1 (IRAK1) also prevents NF-*κ*B activation, induced not by TNF-*α* but also by IL-1*β* and TLR signaling. Taken together, these studies suggest multiple links between OPTN and NF-*κ*B pathway, although not yet clearly characterized for glaucoma. It is important also to point out that OPTN expression is regulated by NF-*κ*B itself by binding to an OPTN promoter and making a negative feedback loop to NF-*κ*B activation [130]. The impact of glaucoma-associated OPTN mutants in this NF-*κ*B negative feedback loop is currently not known.

TBK1 plays a key role in innate immunity by regulating the expression of inflammatory factors, such as NF-*κ*B, IRF3, and IRF7, which has been linked to type-I interferon production [131, 132]. Despite TBK1 immune regulatory capacity, direct inflammatory effects derived from the glaucoma-associated TBK1 duplication have not been documented to date.

## 3. Dysfunction of Neuroglial Cells

Similar to many other neurodegenerative diseases, glial activation is recognized as a hallmark of neuroinflammation in glaucoma. When proinflammatory stimuli arise during injury, astrocytes, Müller cells, and microglia become activated to produce cytokines and chemokines. Although Müller cells, astrocytes, and microglia each have a different developmental origin, they share many functions within the retina, and there is an intricate interrelationship among these cells in the induction of an inflammatory phenotype [133, 134]. However, along with prolonged inflammatory activation of glial cells, there is also a failure in the regulation of immunity, which may ultimately tilt an initial beneficial inflammatory response towards a dysfunctional immune response and neuronal injury.

### 3.1. Astroglia

Astrocytes and Müller cells facilitate the interface between neurons, endothelia, and other glial cells to mediate and modulate metabolic functions, synaptic activity, and homeostasis of the blood-retina barrier [135–138]. These neurosupportive cells also contribute to the defense and homeostasis of neurons by recognizing and responding to local insults. However, under pathological conditions, astrocytes and Müller cells can undergo a pronounced transformation termed gliosis [19, 139–141].

Previous studies revealed that reactive astroglial cells can have both protective and detrimental influences on neuronal survival in glaucoma and other neurodegenerative conditions [19]. Astroglia was shown to become highly reactive in the retina and ONH in glaucomatous human donor eyes, and experimental models of glaucoma [141–143]. Sun et al. demonstrated that a brief period of ocular hypertension may be sufficient to initiate astroglial reactivity in experimental glaucoma [144]. This activation, seen in early stages of the disease, is characterized by morphological alterations and molecular responses that may be detectable even before the damage of RGCs and axons [145]. Although gliosis has the primary aim of circumscribing injured tissues to protect uninjured neurons, astroglial activation may lead to tissue remodeling that result in further biomechanical stress on optic nerve axons and in inadequate metabolic support to RGCs [143–145]. A proinflammatory signature acquired by astrocytes upon stress signals may, therefore, lead to tissue destruction and RGC death [146, 147]. Along with the capacity to trigger innate immunity signaling pathways, reactive astroglia may also compromise the blood-retina barrier, which further increases the access of systemic immune cells into the retina and the optic nerve [137, 145].

### 3.2. Microglia

Microglial cells are myeloid-derived cells that reside in the CNS, providing neurotrophic support and promoting tissue renewal through their phagocytic functions [148]. In inflammatory conditions, microglial cells may also contribute to neuron injury [27, 149, 150]. Despite having a more limited antigen-presentation capacity than that of peripheric professional antigen-presenting cells, microglial cells also act as the first and main form of active immune response in the CNS [138, 148].

Similar to other glial cells, microglia show increased reactivity in the retina and ONH in experimental models and human donor eyes with glaucoma [133, 149, 150]. Microglia react to neural injury with morphological changes, proliferation, migration, and production of inflammatory cytokines that further propagate neuroinflammation. This reaction also includes the release of ROS, NO, and TNF-*α*, leading to neurotoxic effects and aggravated neuronal loss [22, 23, 151]. It has been reported that microglial activation is one of the first events in glaucomatous neural damage occurring prior to RGC loss [152]. In a mouse model of inherited glaucoma (DBA/2J mice), the extent of neurodegeneration correlated with early microglial alterations in vivo [152]. Treatments with minocycline, which inhibit microglial activation, reduced RGC death in the same mouse model [153]. In addition to resident microglia, data from DBA/2J glaucoma model also indicate that glaucomatous proinflammatory state may be amplified by monocytes and other circulating immune cells that invade the ONH and contribute to neurodegeneration [17].

Nitric oxide (NO) is also known to be secreted by microglia in inflammatory conditions [154]. Upregulation of inducible nitric oxide synthase (iNOS) and increased NO levels were found in the ONH of glaucomatous patients and in the retina and ONH of experimental models of glaucoma [155–158]. Inhibition of iNOS by aminoguanidine provided protection to RGCs in a glaucoma model, supporting the possibility of a role of NO in the pathophysiology of glaucoma [159].

## 4. Activation of the Immune System

**Figure 2:**
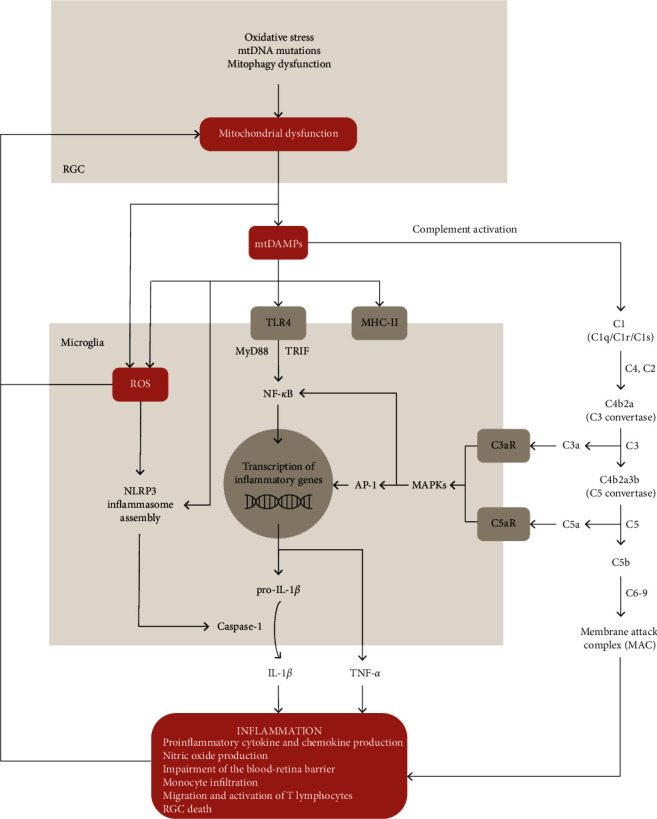
Neuronal mitochondrial damage-associated molecular patterns (mtDAMPs) can act as inducers of chronic inflammation in glaucoma. Mitochondrial-derived components from retinal ganglion cells (RGCs) can trigger inflammatory responses when recognized by complement molecules (classical pathway) and microglial pattern-recognition receptors, such as toll-like receptors (TLRs). TLR signaling induces the transcription of proinflammatory cytokines and chemokines (e.g., pro-IL-1*β* and TNF-*α*) through NF-*κ*B. Inflammation triggered by mtDAMPs can further induce mitochondrial dysfunction, thereby amplifying a vicious cycle of inflammation. In addition to TLR signaling, the presentation of DAMPs through Class II MHC (MHC-II) molecules can further promote the induction of inflammation through T-cell activation. Mitochondrial products can also function directly as NLRP3 activators, which allows caspase-1-dependent release of IL-1*β*. AP-1: activator protein-1; MAPKs: mitogen-activated protein kinases; C3aR: C3a receptor; C5aR: C5a receptor.

Neuronal metabolic dysfunction and reactive gliosis are known to activate glial Toll-like receptor (TLR) signaling and induce complement activation (Figure 2) [20, 160]. In addition to the costimulatory role of ROS in antigen presentation of glial cells, oxidative stress may modify the antigenic features of retina and optic nerve proteins with accumulation of advanced glycation end-products, activation of NF-*κ*B transcriptional program, and stimulation of glial cytokine production in glaucomatous tissues [50, 161, 162].

In addition to the activation of innate immunity in the retina and ONH, glial cells are capable of stimulating systemic immune responses by displaying and releasing signals that favor the recruitment of circulating T lymphocytes. Several factors seem to contribute to the increment of immunogenicity and the activation of inflammatory cues for autoimmunity, namely, an increased exposure of antigens due to neuronal injury, increased expression of immunostimulatory stress proteins, and increased antigenicity due to protein modifications [25, 50, 162–166]. In a similar fashion to peripheral antigen-presenting cells, reactive glia in human glaucoma and animal models display high surface levels of major histocompatibility complex II molecules and stress-associated costimulatory molecules that may enhance antigen presentation to T lymphocytes [161, 167–169].

### 4.1. Release of Damage-Associated Molecular Patterns (DAMPs)

The sterile inflammatory response following glaucomatous damage activates innate immunity mechanisms that are also triggered in infection-induced inflammation. Following infection, microorganisms are initially sensed by pattern-recognition receptors (PRRs) of the innate immune system, which bind conserved molecular patterns that are shared by different classes of microorganisms. These pathogen-associated molecular patterns (PAMPs) include microbial structural components, nucleic acids, and proteins [170]. The list of PRRs that are known to be able to sense PAMPs is extensive and is comprised most notably of four families: TLRs, nucleotide oligomerization domain (NOD)-like receptors (NLRs), C-type lectin receptors (CLRs), and retinoic acid-inducible gene I (RIG-I)-like receptors (RLRs) [170, 171]. PRR ligation triggers multiple signaling pathways that may culminate in the activation of NF-*κ*B, mitogen-activated protein kinases (MAPKs), and interferon regulatory factors (IRFs), which control the expression of proinflammatory cytokines, chemokines, and costimulatory molecules [170–172]. The resulting proinflammatory state is necessary for the generation of a robust antimicrobial response and for the activation of the adaptive immune system.

In addition to recognizing PAMPs, PRRs can be triggered by cellular damage and stress in the absence of microbial infection. Sterile tissue injury and cellular necrosis elicit robust responses characterized by proinflammatory cytokine production and leukocyte recruitment, which are triggered by TLR-, NLR-, and RLR-dependent sensing of “alarmins” or damage-associated molecular patterns (DAMPs) (Figure 2) [170, 173]. DAMPs are endogenous molecules that are isolated within intracellular compartments (e.g., DNA and N-formylated peptides) or are subject to robust metabolism and/or editing in healthy cells (e.g., DNA and double-stranded RNA) [170]. These molecules often exhibit similarities with PAMPs and can be recognized by PRRs during pathological injury [174]. Therefore, this “hidden-self” recognition serves to alert the immune system of cellular or tissue dysfunction.

Due to ancestral bacterial origin, eukaryotic mitochondria maintain prokaryotic features, including a double-membrane structure, the unique cell membrane lipids (e.g., cardiolipin), a circular genome containing CpG DNA, the absence of histones, the ability to replicate independently of the nucleus, the ability to form N-formyl peptides, which are distinct byproducts of mitochondrial translation that reflect their prokaryotic origin, and the use of separate sets of rRNAs and tRNAs encoded by the mitochondrial genome [40, 175]. Thus, cellular damage leading to the release of prokaryotic-like mitochondrial constituents through different modes of cell death can engage PRRs and act as a potent trigger of innate immune responses during stress and injury [40, 175].

Astroglia and microglia are known to participate in early stages of glaucoma but, to date, it is not known which deleterious events contribute the most to the pathogenesis of the disease. A possibility is that stressed or damaged RGCs in the ONH are the seminal source of DAMPs. Heat shock proteins (HSPs), a type of DAMPs, were shown to be upregulated in response to an elevation in IOP and increased in human glaucomatous retinas [20, 138, 176]. More recently, Chen et al. identified both bacterial and host HSPs as possible key natural antigens and demonstrated that commensal microflora induces HSP-specific memory T cells, which are then activated by host HSPs released in the retina after IOP elevation [177]. A second and still valid possibility is that astrocytes and/or microglia initiate gliosis and produce DAMPs independently of RGCs or preceding neuronal dysfunction [178]. Experimental models of glaucoma have identified reactive morphologic changes that precede axonopathy [179–181]. Along with morphologic changes derived from biomechanic causes, neuronal insult in glaucoma may be initiated or aggravated by the absence of the critical glial support, such as in astroglial metabolic substrate transfers (e.g., lactate), neurotrophin secretion, and transforming growth factor-beta (TGF-*β*) production, combined with a detrimental production of TNF-*α* [178, 182]. Speculatively, early glial responses may also constitute a glial effort to guarantee their own survival, with consequential detrimental effects on neuronal support. The identification of initiators of inflammatory events is a relevant area of study in glaucoma.

### 4.2. Activation of Pattern-Recognition Receptors and Inflammatory Signaling

Once stress products are recognized by PRRs such as TLRs, TNF receptor, and inflammasome, specific pathways initiate cascades of events that involve NF-*κ*B (Figure 2) [151]. NF-*κ*B, one of the key regulators of inflammatory immune responses, has been shown to be activated in human glaucoma and in animal models of glaucoma [20, 160].

TLR signaling pathways are among the first to be upregulated in the retinas of patients with glaucoma [19, 20, 183]. Different TLRs display increased expression on astroglia and microglia in human glaucoma, experimental ocular hypertension, and DBA/2J mice with hereditary glaucoma [19, 20]. TLR2, TLR3, and TLR4 were shown to be expressed in microglia and astrocytes of DBA/2J mice retinas, and 11 of 13 TLRs were upregulated in the ONH in early stages of the disease [183]. From these, TLR4 is the most studied Toll-like receptor and is widely expressed in the CNS. Upon DAMPs recognition, TLRs recruit adaptor proteins such as myeloid differentiation primary-response protein 88 (MyD88) and/or Toll/interleukin-1 receptor domain-containing adaptor including interferon-*β* (TRIF) to activate downstream transcription factors. These transcription factors include members of the NF-*κ*B, AP-1, and interferon regulator factor families, which allow the initiation of the transcription of amplifiers and effectors such as TNF-*α*, IL-1*β*, IL-6, and an array of chemokines (e.g., CCL2, CXCL1, and CXCL10) [138, 171, 184].

Another group of PRRs that can modulate microglial inflammatory response is the NOD-like receptors, which form protein complexes commonly known as inflammasomes. Cooperative downstream cross-talks between TLRs and NOD-like receptors can lead to the maturation and release of cytokines like IL-1*β* and IL-18 [185–187]. Mitochondria can directly activate inflammasome signaling. Mitochondria-derived or DAMPs-induced ROS activate the NLR family pyrin domain-containing 3 (NLRP3) inflammasome pathway [188]. NLRP3 is normally associated with the endoplasmic reticulum membrane but, upon activation, is redistributed to nuclear and mitochondrial membranes, where it oligomerizes with apoptosis-associated speck-like protein containing a CARD (ASC) and procaspase-1 to form the NLRP3 inflammasome (Figure 2) [185–187]. NLRP3 inflammasome activation leads to caspase-1-dependent secretion of IL-1*β* and IL-18 and an inflammatory form of cell death termed as pyroptosis [188]. Studies in neurodegeneration in the CNS revealed that mitochondrial dysfunction, including the blockade of mitophagy, is sensed by the NLRP3 inflammasome [188, 189]. Several molecules were specifically identified as DAMPs by these complexes, namely, mtDNA, ATP, and cytochrome C [114, 188]. Despite this evidence, it has not been identified yet how the various aspects of mitochondrial dysfunction converge to a common pathway to activate NLRP3 inflammasome [188, 189]. On the other hand, in bone marrow-derived macrophages, caspase-1 was shown to increase mitochondrial disassembly through the activation of multiple deleterious pathways that amplify ROS production, as well as through the inhibition mitophagy mediated by cleavage of Parkin [190]. NLRP1 and NLRP3 inflammasomes seem to be involved in the pathogenesis of glaucoma in models of acute glaucoma, and, to date, only one study has demonstrated the presence of NLRP3 inflammasome in human glaucomatous eyes [160, 191, 192]. More studies are required to better understand the role of inflammasomes in the progression of glaucoma.

In addition to stress/damage sensors (e.g., TLRs and NLRP3) and inflammatory transducers (e.g., MyD88 and NF-*κ*B), effectors and amplifiers of inflammation are also upregulated in glaucoma. Proteomic analysis of human and experimental glaucomatous retinas revealed an upregulation of kinases that are involved in the activation of the NF-*κ*B pathway, such as RIPK, NIK, and I*κ*K [19, 160]. Activation of NF-*κ*B results in the transcription of IL-1 cytokine family, which can further amplify inflammation by inducing a secondary release of cytokines in microglia and astrocytes. IL-1 cytokines were shown to be upregulated in the ONH at early stages of DBA/2J glaucoma in mice, and antioxidant treatment could afford the downregulation of proinflammatory cytokines and NF-*κ*B [183, 193].

The proinflammatory imbalance seen in glaucomatous tissues is characterized by a marked increase of TNF-*α* production, which is also linked to RGC death [23, 151, 161, 194, 195]. TNF-*α* release can itself affect mitochondrial function by impairing the function of mitochondrial components, reducing ATP production, increasing ROS, and depolarizing the mitochondrial membrane potential [39]. Increased ROS can then have further detrimental effects by maintaining the activation of NF-*κ*B and the production of proinflammatory signaling. The stimulation of neuroinflammation can therefore also have a damaging effect on RGC mitochondrial function itself, thus creating a vicious cycle.

### 4.3. Activation of the Complement System

The complement system is part of the innate immune defense, and it consists of a number of small proteins that provide immune surveillance both peripherally and in the CNS. Complement proteins are expressed in normal physiological processes of the retina and become increased in pathological conditions like inflammation, aging, and trauma and in neurodegenerative diseases such as Alzheimer's disease, Parkinson's disease, and glaucoma [138, 196]. In addition to its role in pathogen recognition and removal, the complement system is also involved in homeostasis by synapse elimination and clearance of potential mediators of damage or injury [197, 198]. Thus, the decisive element that tilts the balance between a homeostatic or a proinflammatory complement-mediated response appears to be the presence of specific danger signals associated with chronic inflammation.

The complement system comprises three distinct and tightly regulated activatory cascades known as the classical, alternative, and lectin pathways [197]. All pathways, which converge in the activation of C3 convertase, contribute to the opsonization of foreign pathogens or apoptotic cell/cellular compartments, the release of inflammatory signaling through anaphylatoxins, and the formation of the membrane attack complex (MAC) [199, 200]. The classical pathway (Figure 2) is initiated when the C1 complex recognizes, among others, antigen-antibody complexes, mitochondrial membrane components, apoptotic cells, or amyloid fibrils [201]. This recognition leads to the formation of C3 convertase and cleavage of C3 protein into C3a and C3b. C3a is an anaphylatoxin that attracts phagocytes to sites of inflammation, and C3b acts as opsonin, which further amplifies complement activation by inducing the cleavage of C5 into C5a and C5b by C5 convertase. C5a is also an anaphylatoxin, and C5b leads to the formation of a lysis-inducing channel in the targeted cell together with C6, C7, C8, and C9 [138, 200].

Increased complement activation has been linked to decreased RGC survival in humans and in animal models of glaucoma, suggesting an involvement of the complement system in the progression of the disease [24, 183, 202–204]. Studies in rodents and nonhuman primates indicated an increased expression of multiple components of the classical complement cascade, which seem to constitute one of the earliest signaling responses to high IOP in the retina and ONH [183, 205, 206]. In DBA/2J glaucomatous mice, an increment of the C1 complex protein C1QA was observed in the ONH before the detection of axonal damage, indicating a potential causal contribution [183]. Deposition of C1QA was also observed in RGC dendrites in glaucomatous retinas of DBA/2J mice, nonhuman primates, and humans, suggesting an involvement of the complement cascade in pathological synapse elimination and/or dendrite remodeling in glaucoma [198, 202–204]. Ablation of C1QA in DBA/2J mice or the viral overexpression of *Crry*, a C3 inhibitor, were both effective in decreasing RGC loss [183, 207, 208].

Downstream components of the complement cascade that are necessary for the formation of MAC seem to also contribute to glaucoma [197]. In human glaucomatous retinas and in experimental models of glaucoma, RGCs were shown to have a marked deposition of MAC [202, 208, 209]. Drug inhibition of complement activation or knockout of the C5 gene reduced MAC deposition as well as RGC loss in rodent models of ocular hypertension and glaucoma [208, 209]. More recently, the intravitreal administration of a monoclonal antibody against the C5 could also afford the preservation of RGCs in an experimental autoimmune model of glaucoma [210]. Together these findings indicate that the complement system can be activated in early stages of RGC injury, which can result in a net increase of retinal damage. Although poorly defined, astrocytes and Müller cells are also able to produce complement proteins and therefore may be conducive to degeneration in a similar fashion [203, 211].

### 4.4. Leukocyte Infiltration

Leukocyte infiltration is a common event in CNS that occurs after injury, disease, and chronic stress [18, 138]. Although early immune responses are likely to be natural attempts to minimize damage after an injury, later immune responses are prone to evolve into chronicity and become more detrimental. In some cases, these beneficial and detrimental events involve molecules in the same pathways [138].

Leukocyte infiltration comprises a cascade of sequential steps that are initiated by the production of cytokines and chemokines in glial cells [23, 138, 212]. The molecular markers involved in leukocyte infiltration include various classes of adhesion/activation/migration proteins expressed on the blood-retina barrier and migrating leukocytes, such as selectins (e.g., P-selectin) and integrin ligands (e.g., VCAM-1 and ICAM-1) on endothelial cells, and selectin ligands (e.g., PSGL1) and integrins (e.g., LFA1) on the surface of leukocytes [18, 138]. These interactions between endothelial cells and leukocytes result in the loosening of endothelial tight junctions to grant access of leukocytes to the CNS [18, 213]. The mechanisms of transendothelial migration in the CNS during injury and disease are very complex, and recent work has shown that the molecules involved in this process are more abundant than previously thought [213].

In the DBA/2J glaucoma model, aberrant upregulation of P-selectin and VCAM-1 was observed in early stages of the disease, suggesting that transendothelial migration of leukocytes takes part in the initiation of glaucomatous damage [17]. Reactive gliosis seems to be able to weaken the perivascular barriers and facilitate access of circulating immune cells and other components such as autoantibodies into the retina and ONH [214]. Breaches in the blood-retina barrier may become visible and result in small optic disc hemorrhages or parapapillary chorioretinal atrophy areas, both of which are commonly detectable in glaucomatous eyes [215, 216]. In line with these results, a longitudinal study revealed a gradual increment of serum autoantibodies in glaucoma patients with optic disc hemorrhages and no detectable change in patients with no optic disc hemorrhages [217]. In addition, the transfer of mononuclear cells from patients with optic disc hemorrhages into immune-deficient mice resulted in increased RGC loss [145, 217]. The tracing of circulating monocytes with an inflammatory profile in the DBA/2J model confirmed the entry of these cells into the ONH and its contribution to glaucomatous damage [17]. These observations are consistent with the higher titers of proinflammatory cytokines found in the blood of patients with glaucoma, as well as the reduction of monocyte infiltration when DBA/2J mice were treated with radiation [17, 218, 219].

Recent findings shed light on the involvement of T-cell-mediated mechanisms in the pathogenesis of glaucoma. This notion is supported by the observation that local inflammation in the retina and ONH is required for T cell crossing of the blood-retina barrier and T cell accumulation is more prominent in inflammatory areas that are more susceptible to high IOP [177, 220–222]. Mice deficient in T cells, but not B cells, displayed a dramatically attenuated RGC and axon damage [177]. One report, however, pointed out that neurodegeneration could be induced in mice, with IOP being within normal or elevated range, after the adoptive transfer of T cells isolated from genetic mouse models of glaucoma [177, 223]. These results suggest that activated T cells of glaucomatous mice are also capable of entering the retina with an intact blood-retina barrier, although probably at a much slower rate or under certain conditions [177, 223]. Studies of blood samples from patients with glaucoma have also detected a shift in the T lymphocyte signature towards a proinflammatory Th1 phenotype and an imbalance of regulatory T cells, indicating a possible lack of efficient T-cell suppression and a minor role of B cells in the disease process [218, 219]. Despite these observations, it remains unclear which subsets of T cells predominate as effector cells or act as initiators of glaucomatous neurodegeneration.

## 5. Conclusion

Numerous forms of endogenous and environmental stressors may disrupt mitochondrial function by impacting mitochondrial homeostasis. Mitochondrial function in RGCs may decline progressively in association with physiologic aging, leading to the release of multiple mitochondrial DAMPs. These misplaced or altered mitochondria-derived molecules may subsequently trigger innate immune responses and result in the onset or progression of inflammatory neurodegenerative diseases, such as in glaucoma. However, the precise mechanism of how mitochondrial DAMPs lead to glaucomatous neurodegeneration is yet to be fully dissected.

There are also mechanisms of glial dysfunction that contribute to neurodegeneration in the early stages of glaucoma. Despite the evident role of inflammation in the disease, to date, it is still not known which exact inflammatory signals lead to activation of glia during the disease progression and how intricate these processes are to neuronal dysfunction, retina-blood barrier impairment, or systemic inflammation. Several experimental challenges hinder a comprehensive understanding of glaucoma. Unlike neurons, microglia and astrocytes are challenging to study in vitro as these cells acquire different phenotypes that hardly resemble in vivo conditions. Several important questions, therefore, remain open, such as, which and how mitochondria-derived molecules contribute to neuroinflammation, and how upstream or downstream this process is in the progression of the disease.

An answer to these questions will facilitate the design of better therapeutic options that are not merely supportive. As glaucoma has many subphenotypes, finding a common therapeutic solution for all poses many challenges. Nonetheless, considering that RGC death is the final common pathway for a very complex pathology, identifying and targeting the key events of neuronal, glial, and immune dysfunction may help halt the neurodegenerative process in the early stages of the disease.
